# Identification of *Salmonella enterica* serovar Kentucky genes involved in attachment to chicken skin

**DOI:** 10.1186/s12866-016-0781-9

**Published:** 2016-07-29

**Authors:** Sanaz Salehi, Kevin Howe, John Brooks, Mark L. Lawrence, R. Hartford Bailey, Attila Karsi

**Affiliations:** 1 Department of Pathobiology and Population Medicine, College of Veterinary Medicine, Mississippi State University, Mississippi State, Mississippi, USA; 2 USDA-ARS, Genetics and Precision Agriculture Unit, Mississippi State, Mississippi, USA; 3 Department of Basic Sciences, College of Veterinary Medicine, Mississippi State University, Mississippi State, Mississippi, USA

**Keywords:** *Salmonella*, Kentucky, Flagella, Poultry, Skin, Attachment

## Abstract

**Background:**

Regardless of sanitation practices implemented to reduce *Salmonella* prevalence in poultry processing plants, the problem continues to be an issue. To gain an understanding of the attachment mechanism of *Salmonella* to broiler skin, a bioluminescent-based mutant screening assay was used. A random mutant library of a field-isolated bioluminescent strain of *Salmonella enterica* serovar Kentucky was constructed. Mutants’ attachment to chicken skin was assessed in 96-well plates containing uniform 6 mm diameter pieces of circular chicken skin. After washing steps, mutants with reduced attachment were selected based on reduced bioluminescence, and transposon insertion sites were identified.

**Results:**

Attachment attenuation was detected in transposon mutants with insertion in genes encoding flagella biosynthesis, lipopolysaccharide core biosynthesis protein, tryptophan biosynthesis, amino acid catabolism pathway, shikimate pathway, tricarboxylic acid (TCA) cycle, conjugative transfer system, multidrug resistant protein, and ATP-binding cassette (ABC) transporter system. In particular, mutations in *S*. Kentucky flagellar biosynthesis genes (*flgA*, *flgC*, *flgK*, *flhB,* and *flgJ*) led to the poorest attachment of the bacterium to skin.

**Conclusions:**

The current study indicates that attachment of *Salmonella* to broiler skin is a multifactorial process, in which flagella play an important role.

## Background

*Salmonella* contamination is an important food safety concern in poultry processing plants. Recently, *Salmonella enterica* serovar Kentucky has been recognized as the most prominent *Salmonella* serovar in poultry processing [1]. According to the National Antimicrobial Resistance Monitoring System (NARMS), the prevalence of *S*. Kentucky isolates from broiler chicken has elevated from 25 % in 1997 to 50 % in 2007 [2]. Although this serovar is not considered a major source of human disease, high incidence of *Salmonella* Kentucky and the emergence of its recent multi-drug resistant strain outside US with high resistance level to ciprofloxacin, indicates this serovar could be a potential threat to public health.

*Salmonella* contamination persists in all stages of chicken processing regardless of the hygienic steps taken. While poultry intestines are considered the most probable origin of contamination, abundant bacteria have been detected on the surface of the broilers. There have been numerous studies on *Salmonella* attachment to chicken skin, however, specific knowledge on the mechanism of attachment is lacking. Bacterial attachment, according to one study, was a result of bacterial retention in a network of fibers that forms when chicken muscle fascia is immersed in water [3]. In another study, *Salmonella* isolation from cervices and feather follicles suggested that the bacterium can be entrapped in water inside the follicles [4]. While cell charge was considered an important attachment factor [5], another study indicated that cell charge did not affect the attachment rate [6]. Bacterial concentration and inoculation time are other aspects that have been suggested as contributors to the attachment of bacterium to chicken skin [7]. There also has been conflicting information on the role of some surface structures (e.g., fimbriae, pili, and flagella) on the attachment of the bacterium to the broiler skin surface [8–10].

Several *Salmonella* surface proteins that appear to mediate adhesion are involved into chicken fascia, which is composed of collagen and elastin fibers interspread in the glycosaminoglycan (GAG) matrix by binding hyaluronan [11]. However, the exact characteristics and properties of these binding sites are not completely understood.

The recent emergence of a ciprofloxacin-resistant strain in a broiler prevalent serovar of *Salmonella* [12] highlights the need to expand our knowledge of the *S*. Kentucky attachment mechanisms broiler skin. The purpose of this current research was to identify *S*. Kentucky genes mediating the bacterial attachment to chicken skin. Identification of *Salmonella* attachment mechanisms to poultry skin could allow development of strategies to reduce carcass contamination during processing, which could assist the broiler processing industry in meeting regulatory concerns for pre- and post-harvest food safety.

## Results

### Identification of mutants with reduced skin attachment properties

**Fig. 1:**
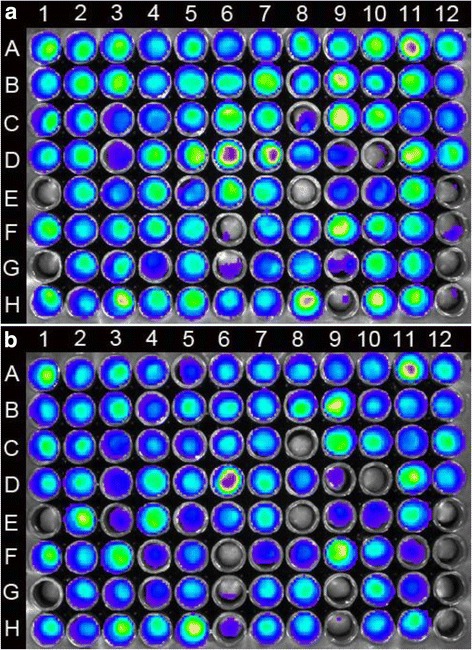
Bioluminescence (p/s/cm^2^/sr) of 96-well plate containing chicken skin cuts: **a** before washing and **b** after washing of chicken skin cuts. The first four wells in the last column (A12, B12, C12, D12) are wild type *S*. Kentucky strain *S*kTn7lux and the last four wells in the same column (E12, F12, G12, H12) are *E. coli* DH5α. The remaining wells are individual mutants that have been replicated in four plates. Examples of mutants with significantly decreased skin binding prior to the main wash are in wells E1, G1, F6, E8, D10 and H9

In total 2,112 *S*. Kentucky mutants were screened for their ability to bind to poultry skin. In the first screening, 264 candidate mutants with decreased bioluminescence relative to wild type *S*. Kentucky strain *S*kTn7lux were identified, including candidate mutants with decreased bioluminescence on chicken skin before and after the 1 h washing step (Fig. 1). Of these candidates, 88 showed a reduction in bioluminescence even before the washing step. The remaining 176 mutants had similar bioluminescence to strain *S*kTn7lux before washing but they displayed reduced bioluminescence after washing with agitation. After the second screening, we identified 66 mutants with decreased bioluminescence on chicken skin compared to strain *S*kTn7lux. Of these, 44 had decreased binding after the final wash step and 22 had decreased binding prior to the final wash. Wild type *S*. Kentucky was not removed from chicken skin after an hour of washing with agitation, while *E. coli* DH5α was completely removed (Fig. 1). A total of 66 mutants showing complete or reduced attachment were chosen for transposon end mapping.

### Identification of transposon insertions in *S.* Kentucky genome

Transposon insertion sites of 66 mutants with attenuated attachment to chicken skin were identified (Tables 1 and 2). Mutants that demonstrated attachment attenuation were classified into two phenotypic groups. The first group showed reduced skin attachment compared to *S.* Kentucky *S*kTn7lux only after 1 h washing with agitation. This group had transposon insertions in various genes: lipopolysaccharide (LPS) biosynthesis, amino acid catabolism, shikimate pathway, TCA cycle, conjugative transfer system (*traD*), signaling and transportation system, phage tail fiber protein H, fimbrial export usher protein, membrane proteins, and several hypothetical proteins (Table 1). The second group had decreased skin attachment prior to 1 h washing. These mutants mostly had transposon insertions in different flagella structural genes (Table 2).

**Table 1:** Skin attachment attenuated mutants removed after washing step

Mutant^a^	Protein ID	Location^b^
P02F10-P01G01	3-dehydroquinate dehydratase	*MAR2xT7*^TActgtccggtggttagcgcctgttcg
P04G08-P01E02	Magnesium and cobalt transport protein CorA	*MAR2xT7*^TAcgcgcaatcgctcgtcgtcgtccgg
P07D05-P01C06	Major outermembrane lipoprotein	*MAR2xT7*^TAaataccggaagtaatagttatcctg
P07G06-P01E06	Dihydrolipoamide acetyltransferase	*MAR2xT7*^TAtgtccgttcaccagaaacagcaaca
P07G09-P01F06	Dihydrolipoamide succinyl transferase	*MAR2xT7*^TAgctttcagtttcgcccgacgtatac
P08F01-P01A08	Poly nucleotide phosphorylase/polyadenylase	*MAR2xT7*^TAagcatggatgacaccgccgtattcg
P08C05-P01B08	Type IV conjugative transfer system coupling protein TraD	*MAR2xT7*^TAccaggaacgtcccaaagtggcgccg
P09B04-P01D09	Lipo polysaccharide biosynthesis protein RffA	*MAR2xT7*^TAtgtaacgtttaagcgcggcggtgtt
P09E05-P01F09	ADP-heptose:LPS heptosyl transferase II	*MAR2xT7*^TAaacgaatttggcaacacccaggcgc
P10H10-P01D10	Anti-terminator-like protein	*MAR2xT7*^TAtattgataaacctcacgcccggcta
P10D11-P01G11	DNA helicase IV	*MAR2xT7*^TAtttgtcccgatcattcaaaacggcg
P04H01-P01F02	Phage tail fiber protein H	*MAR2xT7*^TActcacgtctggaaccaggttaccgg
P06F05-P01H04	Precorrin-4C11-methyl transferase	*MAR2xT7*^TAtgccggttcgctgatcaataccgaa
P10F06-P01B11	NADH pyro phosphatase	*MAR2xT7*^TAtggatcgtataattgaaaaattaga
P10D07-P01G10	Conserved protein with nucleoside triphosphate hydrolase Domain	*MAR2xT7*^TAgtgttcaagcagttgcaccatcgcg
P08F09-P01H07	Oligoribonuclease	*MAR2xT7*^TAtctaaacgcctttaccgatctgaaa
P12F08-P02D02	Glutamyl-Q tRNA (Asp) synthetase	*MAR2xT7*^TAtctccaccgccgcgacggactgttt
P13H05-P02A03	Chaperone protein HscA	*MAR2xT7*^TAtaccaactctctggttgcgacggtt
P14B06-P02H03	Chaperone protein HscA	*MAR2xT7*^TActgatcgtcgggcgcggcggcggtt
P16D03-P02A05	Shikimate 5-dehydrogenase AroDI gamma	*MAR2xT7*^TAcgaagcgctggatctcaattatctc
P16H02-P02C05	Fatty acid oxidation complex sub-unit alpha	*MAR2xT7*^TAcagcgggccgaggtgttgatactgc
P17C05-P02A07	SppA	*MAR2xT7*^TAatgctttatcctcaccaaggtacaa
P18H08-P02C07	NADH dehydrogenase sub-unit H	*MAR2xT7*^TAattgggtggtggccgatttaaacat
P23E10-P02E10	Ribulose-phosphate 3-epimerase	*MAR2xT7*^TAcactttgacgtcatggataatcact
P25D03-P02C11	Tryptophan synthase beta sub-unit	*MAR2xT7*^tgtgccgcagatcctgatgcctgcg
P15C06-P02G04	ATP-dependent RNA helicase DeaD	*MAR2xT7*^TAtaccgattgaagtgggccgtgatgt
P11H11-P02B02	Putative regulatory protein	*MAR2xT7*^TActgtcagcaatggccggaaaaagga
P15C03-P02H04	Glutathione reductase	*MAR2xT7*^TActtcatacgacaacgtgctgggcaa
P21E02-P02C09	Aldolase	*MAR2xT7*^TAtggtgtaatccagcaatttcctggc
P12C04-P02F02	Putative sodium/sulfate transporter, partial	*MAR2xT7*^TAcagaatattggcggcggctttggct
P18C07-P02F07	GTP-binding protein	*MAR2xT7*^TActatcctcgctaaaaacaccgctat
P23F01-P02D10	Ornithine decarboxylase	*MAR2xT7*^TAgttggcctcttgcggattcatactg
P16E01-P02B05	Hypothetical protein STY0758	*MAR2xT7*^TAccagggggactgacggcctgtgcag
P19F07-P02H08	Oxidoreductase	*MAR2xT7*^TAtattgagtcctcttccggcgtttcg
P25G02-P02F11	Intramembrane serine protease GlpG	*MAR2xT7*^TAtatatactgtattttgtatgga
P19A07-P02A08	Fimbrial outer membrane usher protein	*MAR2xT7*^TAcgttcggttcaatagcggtttcaat
P23C06-P02B10	Pyruvate dehydrogenase sub-unit E1	*MAR2xT7*^TAcatcaacactattgccgttgaagac
P20C11-P02B09	Alpha ribazole-5'-P phosphatase	*MAR2xT7*^TAcaaataatcatacagtcggacgata
P18D02-P02G07	4-hydroxythreonine-4-phosphate dehydrogenase	*MAR2xT7*^TActctgctaggtgctgcccgacccgg
P22G01-P02A10	Permease protein SitC	*MAR2xT7*^TAagccatgcgcccagaaaactggtca
P13B03-P02E03	Putative sensor kinase protein	*MAR2xT7*^TAcaacaagaaatcgccgagcgcggac
P10C09-P02C01	Exoribonuclease II	*MAR2xT7*^TAtaaccagtcgccgacatcgcgctcc
P22E10-P02G09	Phosphorpyruvate hydratase	*MAR2xT7*^TAtcacaccaggcacagccgaccggac
P19H03-P02B08	High-affinity zinc transporter periplasmic protein	*MAR2xT7*^TAaaaccacgcgtacaagcgttgactt

^a^Mutants are listed according to the degree of attachment attenuation

^b^ *MAR2xT7*, mariner transposon; ^, insertion point; TA, two-base TA duplication; lowercase letters, 25-bp flanking unique gene sequences of *S. enterica*

**Table 2:** Attachment attenuated mutants removed before the washing step

Mutant^a^	Protein ID	Location^b^
P09G05-P03F06	Flagellar basal-body P-ring formation protein FlgA	*MAR2xT7*^TAttcatcgcctgaccttccgcattga
P03G04-P03G01	Flagellar basal-body rod protein FlgC	*MAR2xT7*^TAgctgcgcaggctgacatcgtgttg
P24B04-P03D10	Unnamed protein product	*MAR2xT7*^TAttcccctggatgattttttacgcag
P21C09-P03G09	Flagellar biosynthesis protein FlhB	*MAR2xT7*^TAttccgtggcgctgcagtatgacgaa
P05D08-P03D03	Multidrug resistance protein, SMR family	*MAR2xT7*^TAcgcggcttaaaagggccaattcccg
P05H05-P03C03	Cysteine/glutathione ABC transporter membrane/ATP-binding comp.	*MAR2xT7*^TAgttaaaactgtaaattcccgcgaag
P09H05-P03G06	Lipopolysaccharide core biosynthesis protein	*MAR2xT7*^TAgcctgttctgggcgctgacagaaga
P15B11-P03C08	Flagellar hook-associated protein Flgk	*MAR2xT7*^TAgcaacagtaataatgccgataaaac
P22D04-P03H09	tRNAuridine5-carboxymethylaminomethyl modification enzyme GidA	*MAR2xT7*^TAacgaatcacgtcatgggttttctca
P25E10-P03E10	Flagellar rod assembly protein/muramidase FlgJ	*MAR2xT7*^TAcgttatagctgggttcgccattctc
P16F02-P03F8	DamX protein	*MAR2xT7*^TAtttgccgcacatgctgcgagataaa
P13F05-P03G07	NitrogenregulationproteinNR2, partial	*MAR2xT7*^TAcgtggcgcggcgcagctgcagagca
P05E06-P03F02	Dimethyl adenosine transferase	*MAR2xT7*^TAtttatcagcaggacgccatgaccat
P17E05-P03D09	1-acyl-glycerol-3-phosphateacyltransferase	*MAR2xT7*^TAgaatgccgggctcttaggccttcag
P12H05-P03F07	Chain A, DNA-binding transcriptional repressor Acrr	MAR2xT7^TAagcaacgcgatggcgcgtaaaacca
P25F11-PO3C11	Cystathionine beta-lyase	*MAR2xT7*^TAtatgaccagccgcggtctgcgcaca
P08C02-P03E05	Flagellar basal body P-ring biosynthesis protein FlgA	*MAR2xT7*^TAttcatcgcctgaccttccgcattga
P13C07-P03H07	ParB gene product	*MAR2xT7*^TAcgactaaactcataagttaacgtac
P02E02-P03B01	Two-component sensor kinase SsrA	*MAR2xT7*^TActtcgagtatggctggataaaacaa
P16F04-P03G08	Hemelyase sub-unit NrfE	*MAR2xT7*^TAtagcccgccagtaccacctgctgac
P06D02-P03F04	Hypothetical proteinSeI_A3977	*MAR2xT7*^TAaacactcaaaacgtcttggtattcg
P05F11-P03H02	Membrane protein suppressor for copper sensitivity ScsD	*MAR2xT7*^TAtaccgtgtcgggcgccggacattct

^a^Mutants are listed according to the degree of attachment attenuation

^b^ *MAR2xT7*, mariner transposon; ^, insertion point; TA, two-base TA duplication; lowercase letters, 25-bp flanking unique gene sequences of *S. enterica*

## Discussion

In the current study, we used random transposon mutagenesis on bioluminescent *Salmonella* Kentucky strain *S*kTn7lux, to identify genes involved in attachment to chicken skin. Our results showed that *S*. Kentucky attachment to broiler skin is a multifactorial process requiring the expression of many genes. We identified two different phenotypic groups of mutants with decreased attachment on chicken skin. We expected to identify *S*. Kentucky mutants that were more susceptible to removal by washing, which mimics the washing process that occurs during the poultry processing. However, we also identified a group of mutants with more severe attachment defect, which had decreased skin binding after simple flushing with a pipette. Notably this second group included six mutants with transposon insertions in flagella genes highlighting the role of flagella in *S*. Kentucky attachment to poultry. These six mutants harbored two different transposon insertions in *flgA*, which encodes flagellar basal-body p-ring formation protein. Other mutated flagellar genes encode flagellar basal-body rod protein (*flgC*), flagellar hook-associated protein (*flgK)*, a rod assembly protein (*flgJ*), and flagellar biosynthesis protein (*flhB*). Attachment defects in flagellar basal body protein mutants suggest that flagellar rotation contributes to skin attachment.

Previous studies have reported conflicting results on the role of flagella on attachment of *Salmonella* to broiler skin. In one study, attachment to broiler skin was dependent on the presence of flagella [10]. In later studies, it was concluded that under controlled conditions, non-flagellated bacteria attached as well as flagellated bacteria [9]. Similar to our results, attachment was found to be a complex reaction, and fimbria and flagella both contribute to the process [8].

Other attachment-defective mutations were in transporter and signaling systems, which have higher expression in attached bacteria, and may work as an efflux pump to help the bacterium resist environmental stress [13]. Thus mutation in these genes may make these mutants more susceptible to environmental conditions. In the current study, attachment-deficient mutant P07D05-P01C06 had an insertion in *lpp* which encodes the major outer membrane lipoprotein. Under specific environmental conditions, an *E.coli* outer membrane lipoprotein, NIp*E*, senses and generates an adhesion signal to the Cpx pathway that leads to stable adhesion [14]. Mutant P09H05-P01G06 had an insertion in the *waaG* gene, which encodes a LPS core biosynthesis protein. In *E. coli*, LPS is known to contribute to attachment. Some mutations in *E. coli* genes encoding lipopolysaccharide core biosynthesis enzymes showed decreased adhesion to solid surfaces [15]. In addition to *waaG*, two other mutants had insertions in LPS biosynthesis genes in the current study: P09B04-P01D09 was mutated in *rffA* which encodes a LPS biosynthesis protein; and P09E05-P01F09 was mutated in *rfaF*, which encodes ADP-heptose: LPS heptosyltransferase II that contributes to synthesis of the inner core backbone of LPS.

P25D03-P01C11 had an insertion in *trpB*, which encodes tryptophan beta sub-unit synthase. Tryptophan is a major factor in forming *Salmonella enterica* serovar Typhimurium biofilms on food surfaces [16]. Amino acids metabolites, especially those involved in tryptophan biosynthesis are up-regulated at early stages of attachment. Although attachment of *Salmonella* to broiler skin is not considered a biofilm formation process, it is comparable with bacterial attachment to solid surfaces at an early stage of biofilm formation. In *E. coli*, over-expression of tryptophan biosynthesis and increased production of tryptophan and its precursor, indole, prepares the bacteria for nutrient-poor environments and increases catabolism of amino acids. Indole also up-regulates detoxifying genes (e.g., drug exporters) to make the bacterium more resistant to toxic compounds and increases bacterial adherence to surfaces [16]. These characteristics can be vital in *Salmonella* adherence to chicken skin. Also, P16D03-P01A05 and P02F10-P01G01 had insertions in *aroDI* and *aroD* which encode 5-dehydrogenase gamma and 3-dehydroquinate dehydratase, respectively. These compounds are both enzymes in the Shikimate pathway and are involved in the biosynthesis of aromatic amino acids. These mutations also emphasize the importance of tryptophan in the attachment process.

## Conclusions

Bioluminescence mutant screening of *S*. Kentucky was applied to identify mutants that are defective in attachment to chicken skin. Results indicate that flagella have an important role in attachment of *S*. Kentucky to broiler skin. Some other pathways that are important for skin adherence include LPS biosynthesis, aromatic amino acid biosynthesis, outer membrane lipoprotein, and transport/secretion systems. Further investigations, especially in flagella structure and basal body genes, could lead to a better understanding of the exact molecular mechanism of *Salmonella* attachment to poultry skin.

## Methods

### Bacterial strains, plasmids and growth conditions

*Escherichia coli* SM10λ*pir* [17] was used as the donor strain in conjugations for transfer of p*MAR2xT7* [18] into bioluminescent *S*. Kentucky strain *S*kTn7lux [19] originally isolated from a broiler processing plant [20]. Bioluminescent *E. coli* DH5α (Thermo Fisher Scientific, Waltham, MA) and bioluminescent wild type *S*. Kentucky strain *S*kTn7lux [19] were used as controls. *E. coli* and *Salmonella* strains were grown on Luria-Bertani (LB) broth and agar plates at 37 °C. Gentamicin and streptomycin added to LB agar plates at 50 μg/ml^−1^ as appropriate.

### Construction of transposon insertion library

p*MAR2xT7* was transferred from *E. coli* SM10λ*pir* into bioluminescent *S*. Kentucky strain SkTn7lux by conjugal mating [21]. Briefly, a colony of *E. coli* SM10λ*pir* carrying p*MAR2xT7* and bioluminescent *S.* Kentucky *S*kTn7lux were inoculated in 5 ml of LB broth at 37 °C overnight at 200 rpm. Equal amounts of overnight culture of donor and recipient (1.5 ml) were pelleted separately by centrifugation, washed three times with LB broth, and then re-suspended in 1 ml of LB broth. Donor and recipient strains were mixed in a 1:3 ratio based on their volume. The mixture was centrifuged at 12,100 x g for 2 min. The harvested cells were diluted in 10 μl of LB broth and transferred to a 0.45 μm sterile filter paper, which was placed on LB agar and incubated at 37 °C for 18 h. The filter was washed with 5 ml LB broth, and 50 μl of the washed bacteria was spread on the LB agar containing gentamicin and streptomycin. Bioluminescence of colonies on agar plates were confirmed using an IVIS 100 Imaging System. A batch of gentamicin-resistant colonies was tested for random transposon insertion using single-primer PCR [22] and sequencing. More than 2,000 colonies of mutant *S*. Kentucky were picked using a pipette tip and inoculated in 150 μl of LB plus gentamicin broth in 96-well plates and incubated in an incubator shaker overnight. Plates were sealed, and the mutant library was stored in 20 % glycerol at −80 °C [21].

### Chicken skin attachment assay

In our previous studies, a chicken skin attachment assay was established, which indicated that the number of attached bacteria to chicken skin can be measured by the bioluminescence intensity of the correlated bacteria [23]. In this work, the chicken skin attachment assay was performed twice. Primary screening was accomplished with one replicate for a total of 2,112 mutants. Those mutants showing reduced attachment went through the final skin attachment assay with four replicates. Each 96-well plate contained four replicates of bioluminescent wild type *S*. Kentucky strain *S*kTn7lux and *E. coli* DH5α. Plates were covered with Breath-Easy film (Diversified Biotech, Boston, MA) and grown at 37 °C overnight at 250 rpm on a shaker incubator. The OD and bioluminescence of each well were measured to ensure the growth and bioluminescence of each mutant. Five microliters from overnight cultures were used to inoculate fresh 96-well plate containing 100 μl LB broth, which were incubated at 37 °C for 2 h to reach the log phase.

The chicken skin was obtained from a commercial broiler processing plant inspected by USDA Food Safety Inspection Service. Chicken skins were cut into uniform, circular sections by 6 mm skin biopsy punch and placed into clear-bottomed 96-well black cell culture plates. 100 μl of log phase mutant culture with known OD and bioluminescence were added to each well and after a brief spin, plates were incubated at 25 °C for 1 h to allow bacterial attachment to the broiler skin. Following incubation, bacterial suspensions were removed by vacuum suction, and the wells were washed with 200 μl of distilled water by pipetting twice to remove unattached bacteria. Plates were incubated at 37 °C for 10 min. and bioluminescent imaging was recorded for15 s of exposure at 37 °C with an IVIS 100 Imaging System. Bioluminescence was quantified using Living Images software as described [19].

To determine the effect of washing on bacterial attachment properties, plates were filled with 200 μl of water and placed in a rotating platform incubator at 700 rpm for 1 h. After the removal of excess solution, bioluminescence on skin sections was measured and recorded for 15 s of exposure. This stage was considered as the main washing step.

### Determination of mutants with attenuated attachment

Bioluminescence (p/s/cm^2^/sr) was measured twice in each assay: prior to and after the final 1 h washing step. In the primary screening, percent bioluminescence reduction was calculated from each mutant, which were then ranked from highest to lowest reduction. In the secondary screening, mutants with highest bioluminescence reduction went through another skin attachment assay with four replicates. Mutants were considered deficient in attachment if their attachment percentage fell out of the lower 95 % confidence limit calculated from attachment rate of wild type strain *S*kTn7lux replicates. Mutants with decreased attachment either before or after the final wash were chosen for transposon end mapping.

### Identification of transposon insertion site

Transposon insertion sites for the 66 mutants with reduced attachment to chicken skin were identified by overlap extension PCR [24]. Briefly, genomic DNA was prepared from overnight cultures using a Wizard Genomic DNA Purification Kit (Promega, Madison, WI). Each 25 μl of PCR contained 0.2 μM forward or reverse transposon specific primer (MAr2xT7F: TACAGTTTACGAACCGAACAGGC or MAR2xT7R TCTATACAAAGTTGGGCATACGG) 0.2 mM dNTPs, 1.5 mM MgCl_2_ and 1.25 U of *Taq* polymerase (Promega, Madison, WI). The PCR was performed using a PTC-100 thermocycler (MJ Research, Water town, MA) with the following cycling steps: initial denaturation (2 min at 94 °C) followed by 25 cycles of denaturation (30 s at 94 °C), annealing (30 s at 55 °C), and elongation (3 min at 72 °C) followed by 30 cycles of denaturation (30 s at 94 °C), annealing (30 s at 30 °C), and elongation (2 min at 72 °C) followed by 30 cycle of denaturation (30 s at 94 °C), annealing (30 s at 55 °C), and elongation (2 min at 72 °C). A final extension of 10 min at 72 °C was also applied. PCR products were cleaned with ExoSAP-IT enzyme mix (USB Corp. Cleveland, Ohio) and used as template in sequencing reactions using BigDye Terminator v1.1 and 0.5 μM of a nested transposon specific primer (MAR2xT7FSeq: GGACCGAGATAGGGTTGAGTG or MAR2xT7R3Seq: AACAATTCGTTCAAGCCGAGA). Transposon specific sequences were trimmed and the remaining sequences were checked against the protein database of the National Center for Biotechnology Information (NCBI) using BLAST+, which revealed the location of transposon insertion [21].

## Abbreviations

ABC, ATP-binding cassette; *flg*, flagellar; GAG, glycosaminoglycan; LPS, lipopolysaccharide; NARMS, National Antimicrobial Resistance Monitoring System; TCA, tricarboxylic acid.

## References

[1] Vasil ML (2007). How we learnt about iron acquisition in Pseudomonas aeruginosa: a series of very fortunate events. Biometals.

[2] Foley SL, Nayak R, Hanning IB, Johnson TJ, Han J, Ricke SC (2011). Population dynamics of Salmonella enterica serotypes in commercial egg and poultry production. Appl Environ Microbiol.

[3] Thomas C, McMeekin T (1981). Attachment of Salmonella spp. to chicken muscle surfaces. Appl Environ Microbiol.

[4] Kim K, Frank J, Craven S (1996). Three‐dimensional visualization of Salmonella attachment to poultry skin using confocal scanning laser microscopy. Lett Appl Microbiol.

[5] Dickson JS, Koohmaraie M (1989). Cell surface charge characteristics and their relationship to bacterial attachment to meat surfaces. Appl Environ Microbiol.

[6] Kim K, Lillard H, Frank J, Craven S (1996). Attachment of Salmonella typhimurium to poultry skin as related to cell viability. J Food Sci.

[7] Kim K, Frank J, Craven S (1996). Attachment of Salmonella on modified poultry skin surface. J Food Sci.

[8] Lillard H (1986). Role of fimbriae and flagella in the attachment of Salmonella typhimurium to poultry skin. J Food Sci.

[9] Lillard H (1985). Bacterial cell characteristics and conditions influencing their adhesion to poultry skin. J Food Prot.

[10] Notermans S, Kampelmacher E (1974). Attachment of some bacterial strains to the skin of broiler chickens. Br Poultry Sci.

[11] Sanderson K, Thomas C, McMeekin T (1991). Molecular basis of the adhesion of Salmonella serotypes to chicken muscle fascia. Biofouling.

[12] Le Hello S, Hendriksen RS, Doublet B, Fisher I, Nielsen EM, Whichard JM, Bouchrif B, Fashae K, Granier SA, Jourdan-Da Silva N (2011). International spread of an epidemic population of Salmonella enterica serotype Kentucky ST198 resistant to ciprofloxacin. J Infect Dis.

[13] Svensson S, Frirdich E, Gaynor E (2008). Survival strategies of Campylobacter jejuni: stress responses, the viable but nonculturable state, and biofilms. Campylobacter.

[14] Otto K, Silhavy TJ (2002). Surface sensing and adhesion of Escherichia coli controlled by the Cpx-signaling pathway. Proc Natl Acad Sci.

[15] Genevaux P, Bauda P, DuBow MS, Oudega B (1999). Identification of Tn10 insertions in the rfaG, rfaP, and galU genes involved in lipopolysaccharide core biosynthesis that affect Escherichia coli adhesion. Arch Microbiol.

[16] Hamilton S, Bongaerts R, Mulholland F, Cochrane B, Porter J, Lucchini S, Lappin-Scott H, Hinton J (2009). The transcriptional programme of Salmonella enterica serovar Typhimurium reveals a key role for tryptophan metabolism in biofilms. BMC Genomics.

[17] Miller VL, Mekalanos JJ (1988). A novel suicide vector and its use in construction of insertion mutations: osmoregulation of outer membrane proteins and virulence determinants in *Vibrio cholerae* requires *toxR*. J Bacteriol.

[18] Liberati NT, Urbach JM, Miyata S, Lee DG, Drenkard E, Wu G, Villanueva J, Wei T, Ausubel FM (2006). An ordered, nonredundant library of Pseudomonas aeruginosa strain PA14 transposon insertion mutants. Proc Natl Acad Sci U S A.

[19] Howe K, Karsi A, Germon P, Wills RW, Lawrence ML, Bailey RH (2010). Development of stable reporter system cloning luxCDABE genes into chromosome of Salmonella enterica serotypes using Tn7 transposon. BMC Microbiol.

[20] Volkova VV, Bailey RH, Rybolt ML, Dazo-Galarneau K, Hubbard SA, Magee D, Byrd JA, Wills RW (2010). Inter-relationships of Salmonella status of flock and grow-out environment at sequential segments in broiler production and processing. Zoonoses Public Health.

[21] Karsi A, Gülsoy N, Corb E, Dumpala PR, Lawrence ML (2009). High-throughput bioluminescence-based mutant screening strategy for identification of bacterial virulence genes. Appl Environ Microbiol.

[22] Karlyshev AV, Pallen MJ, Wren BW (2000). Single-primer PCR procedure for rapid identification of transposon insertion sites. Biotechniques.

[23] Karsi A, Howe K, Kirkpatrick TB, Wills RW, Bailey RH, Lawrence ML (2008). Development of bioluminescent Salmonella strains for use in food safety. BMC Microbiol.

[24] Horton RM, Hunt HD, Ho SN, Pullen JK, Pease LR (1989). Engineering hybrid genes without the use of restriction enzymes: gene splicing by overlap extension. Gene.

